# Unique Genetic and Epigenetic Alterations in Glioblastoma Long‐Term Survivors: Insights From Two Clinical Cases

**DOI:** 10.1111/jcmm.70771

**Published:** 2025-10-14

**Authors:** Elena Anghileri, Evelina Miele, Sara Patrizi, Sabina Barresi, Elisabetta Lazzarini, Luisa Maddaloni, Monica Patanè, Lucia Pedace, Rosina Paterra, Antonio Silvani, Franco Locatelli, Stefano Indraccolo, Bianca Pollo

**Affiliations:** ^1^ Neuro‐Oncology Unit Fondazione IRCCS Istituto Neurologico Carlo Besta (FINCB) Milan Italy; ^2^ Department of Onco‐Hematology, Cell Therapy, Gene Therapies and Hemopoietic Transplant Bambino Gesù Children's Hospital, IRCCS Rome Italy; ^3^ Pathology Unit Bambino Gesù Children's Hospital, IRCCS Rome Italy; ^4^ Basic and Translational Oncology Unit Istituto Oncologico Veneto (IOV)‐IRCCS Padua Italy; ^5^ Neuropathology Unit Fondazione IRCCS Istituto Neurologico Carlo Besta Milan Italy; ^6^ Department of Surgery Oncology and Gastroenterology University of Padua Padua Italy

**Keywords:** chromothripsis (CT), DNA methylation (DNAm) profile, glioblastoma (GBM), long‐term survival (LTS), Lynch syndrome (LS), *MSH6* mutation, very LTS (VLTS)

## Abstract

The biological mechanisms driving the long survival in glioblastoma (GBM). Five‐year long‐term survival (LTS) and 10‐year survival very long‐term survival (VLTS) remain significantly understudied. Here we molecularly detailed two cases. AR10‐046 (VLTS) was affected by a giant cell‐GBM, classified as the pedHGG_RTK1a subtype according to the v12.5 Heidelberg brain tumor methylation classifier. Somatic and germline MSH6 mutations, typically in Lynch syndrome, and high tumour mutational burden were detected. The copy number variation plots showed chromosome 1q gain and chromosome 13 loss with no other typical GBM alterations. AR10‐037 (LTS) suffered from a classical GBM, identified as pedHGG_MYCN subclass. Apart from the canonical chromosome 7 gain and chromosome 10q loss, we observed MDM2 gene amplification and possible rearrangements on chromosome 12 and 18 with the typical aspect of chromothripsis, harbouring two putative new gene fusions: CPSF6::CPM and PTPRR::RAB3IP. We described two patients with peculiar tumour molecular profile, widening the scenario of clinical and molecular variability in such patients.

Glioblastoma (GBM) stands as the predominant primary brain tumour, with a 5‐year survival rate of 7% and a 10‐year survival rate of 4.7%, according to Ostrom [1]. The recent 2021 World Health Organization (WHO) Central Nervous System (CNS) Classification (CNS5) stated that GBM needs to have IDH1/2 wild type; moreover, it introduced the concept of molecular diagnosis of GBM based on *TERT* promoter mutation, *EGFR* amplification, and +7/−10 copy number changes in IDH‐wildtype diffuse astrocytomas: overall, based on such definition, probably the rates of long‐term survival for GBM are even lower compared to what was reported before 2021.

The designation of long‐term survival (LTS) and very long‐term survival (VLTS) in GBM typically denotes patients surpassing the 5‐and 10‐year mark, respectively. Despite this distinction, the underlying biological mechanisms driving such prolonged clinical outcomes remain significantly understudied.

As we recently published, specific methylation classes according to the very last version of the Heidelberg brain tumor classifier (BTC) as well as an overall global higher methylation can be crucial determinants of less aggressive brain tumours [2].

In the present study, we describe in detail one VLTS (AR 10‐046) and one LTS (AR 10‐037) case out of those previously reported [2], based on the peculiarity of their molecular profile.

## 1 Case Reports

**FIGURE 1:**
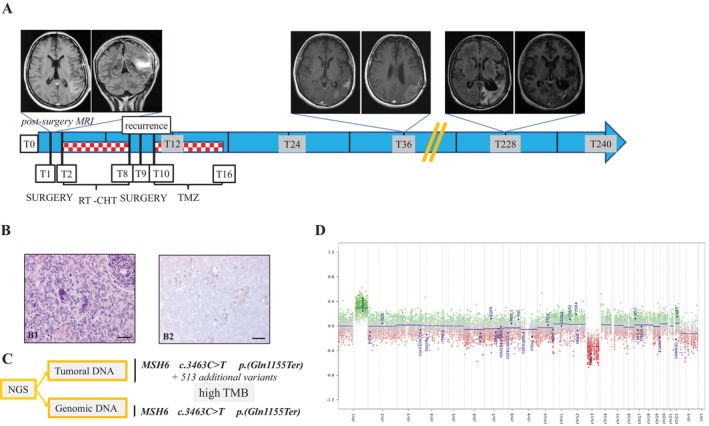
Clinical, radiological, histopathological, genetic and epigenetic features of case AR 10‐046. (A) Clinical timeline and magnetic resonance (MR) imaging. Two months after symptoms onset (T1) the patient underwent gross total resection, as confirmed by post‐surgical axial and coronal MR images. Adjuvant therapy was administered, but tumour recurrence occurred at 8 months, prompting a second surgical resection. Histology showed a gcGBM. Following complete resection, the patient received six cycles of adjuvant temozolomide and continued with routine clinical and radiological follow‐up. Sustained disease stability is illustrated by axial MR scans at T36 and T228. (B) Histopathological features from the initial surgery. Haematoxylin and eosin staining (B1) and MSH6 IHC (B2) reveal loss of MSH6 expression in tumour cells, with retained expression in non‐neoplastic cells (e.g., endothelial cells). Scale bar = 50 μm. (C) Summary of genetic testing results from tumour and germline DNA. (D) CNV plots inferred from DNA methylation (DNAm) data of the case AR 10‐046. The plot illustrates chromosomal alterations across autosomes and sex chromosomes (X/Y), with gains and amplifications indicated by positive deviations (green) and losses by negative deviations (red) from the baseline. Twenty‐nine genomic regions of known relevance to tumour biology are specifically highlighted.

Patient AR 10‐046 was a 31‐year‐old female diagnosed with a left frontoparietal lesion treated by surgery: the histology showed features suggestive of giant cell GBM (gcGBM). The patient was then treated with conformational radiotherapy (with a total dose of 60 Gy in 30 fractions) and cisplatin and carmustine as a compassionate use programme. After 6 months from the diagnosis, she experienced local tumour recurrence and underwent lesion exeresis and adjuvant temozolomide, maintaining disease stability until the last follow‐up on 1 August 2024 for a total of 243 months (Figure 1A). Histology confirmed the diagnosis of gcGBM, with some inflammatory lymphoid infiltrates (Figure 1B1). According to the v12.5 BTC, the tumour was assigned to the methylation subclass pedHGG_RTK1a subtype (score > 0.99), frequently associated with Lynch syndrome (LS). Indeed, we detected *MSH6* mutation [c.3463C>T; p.(Gln1155Ter)] in tumour DNA together with high tumour mutational burden (TMB) (Figure 1C) and negative staining for MSH6 protein (Figure 1B2). Thus, we searched for the germline variant and confirmed LS diagnosis (Figure 1C). Interestingly, the copy number variation (CNV) plots showed chromosome 1q gain and chromosome 13 loss with no other typical GBM alterations (Figure 1D). Moreover, although no copy number loss was observed at the MSH6 locus (2p16.3), we found an additional nonsense mutation in MSH6 [c.2539G>T; p.(Glu847Ter)] at low confidence which could represent a second hit in the MSH6 gene, fitting the Knudson two‐hit hypothesis for tumour suppressor genes.

**FIGURE 2:**
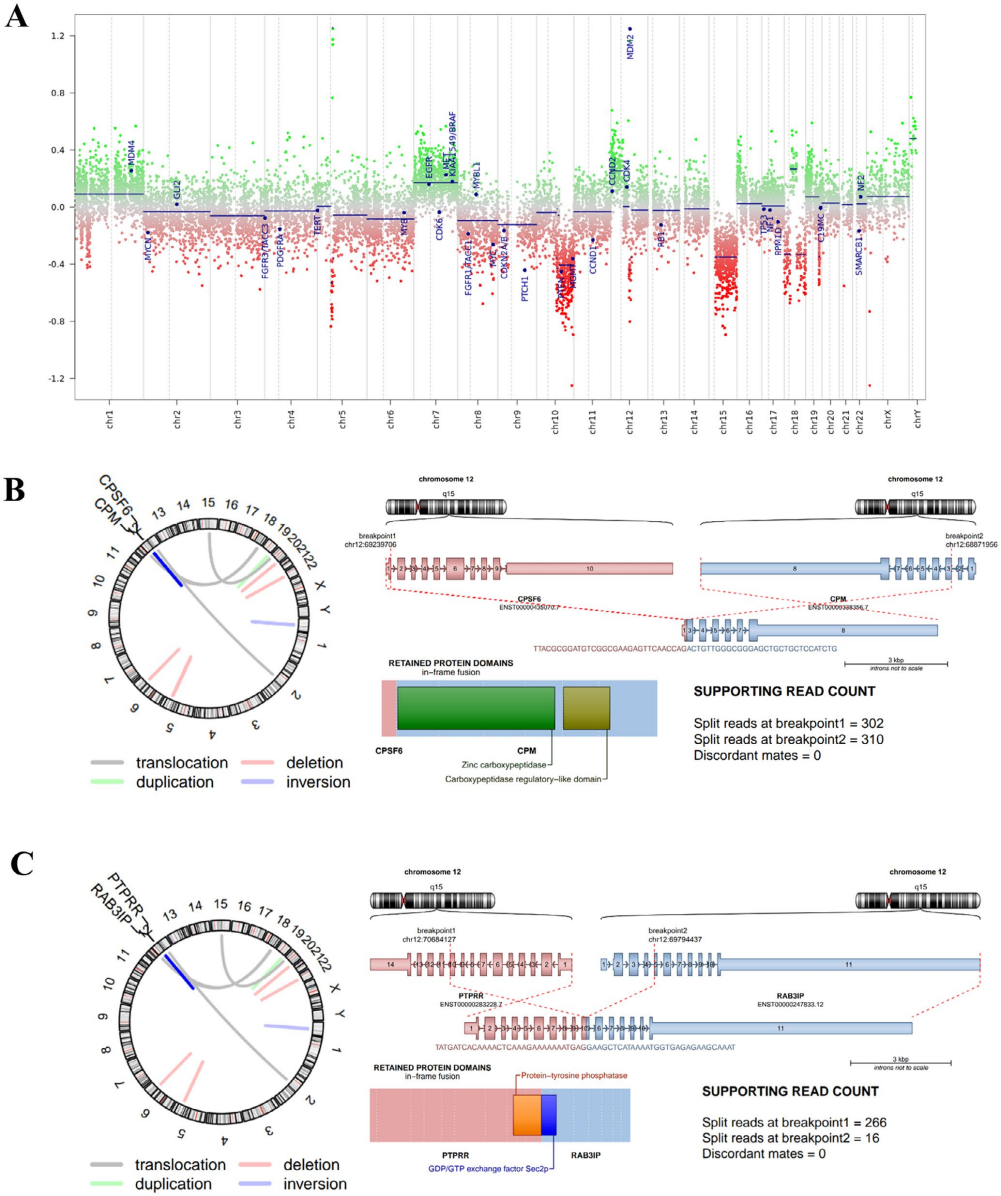
Genetic and epigenetic profile of case AR 10‐037. (A) CNV plots inferred from DNAm data of the case AR 10‐037 illustrating chromosomal alterations across autosomes and sex chromosomes. As in Figure 1D, gains/amplifications are shown as positive deviations (green) and losses as negative deviations (red) from the baseline. Twenty‐nine tumour‐relevant genomic regions are highlighted. (B, C) Representation of structural rearrangements on chromosome 12 showing the putative fusions (B) *CPSF6::CPM* (chr12:69239706:+, chr12:68871956:−) and (C) *RAB3IP::PTPRR* (chr12:69795344:+, chr12:70701323:−). The cartoons depict the exact structural rearrangements with the breakpoints on chromosome 12 of the top high‐confidence and in‐frame fusion transcripts with indicated the retained protein domains and the supporting read counts.

Patient AR 10‐037 was a 70‐year‐old male, clinically self‐sufficient (Karnofsky Performance Score: 70) at the disease onset, affected by a left temporal lobe GBM undergone to surgery. He survived 73 months after GBM diagnosis. Histology was classical GBM. According to the v12.5 BTC, DNA methylation (DNAm) profiling yielded a calibrated score of 0.5, tending towards the pedHGG_MYCN subclass; however, this score is non‐diagnostic and did not match any reference methylation class. At CNV examination, apart from the canonical chromosome 7 gain and chromosome 10q loss, we observed *MDM2* gene amplification and possible rearrangements on chromosomes 12 and 18 with the typical aspect of chromothripsis (CT) (Figure 2A). To identify novel gene fusions within CT events, we performed RNASeq analysis confirming several genomic rearrangements, many of which involved chromosome 12. Considering only high‐confidence and in‐frame fusion transcripts (*n* = 7), the candidate events were two intergenic deletions on chromosome 12 resulting in two novel in‐frame rearrangements *CPSF6*::*CPM* and *PTPRR*::*RAB3IP* (Figure 2B), never reported in brain tumours.

## 2 Discussion

As is well known, both clinical‐radiological variables and histological‐molecular features influence long‐term survival in GBM patients. Among others, progression free survival (PFS) and overall survival (OS) are strongly correlated [3]. In particular, PFS and treatment post‐Stupp protocol [4] were reported as positive prognostic factors, and both were not present in our case AR 10‐046. About other GBM features, the role of GBM location on prognosis is still debated [2]. Regarding the histological subtype, recently it was reported that gcGBM accounts for around 2% of all IDHwt GBM and had better survival than conventional GBM, but without reaching statistical significance [5, 6, 7]. AR 10‐046 was diagnosed as LS, bearing a germline *MSH6* mutation, the third most common mismatch repair (MMR) mutation in LS [8]. Although no copy number loss was observed at the MSH6 locus (2p16.3), an additional somatic inactivating variant was identified, in line with Knudson's second‐hit hypothesis. Histology showed gcGBM with a high tumour mutational burden as described by others [7, 8]. Such a profile of hypermutated characteristics with a pathogenic variant in the MSH6 gene is consistent with mismatch repair deficiency. This may have contributed to an elevated neoantigen load and enhanced immunogenicity, potentially facilitating immune surveillance and tumour control.

Immune checkpoint inhibitors (ICIs) (pembrolizumab or nivolumab) can represent a promising therapy, for selected cases reported as long [8] or very long‐term [9] responders. A recent study involving 459 GBM patients identified de novo mismatch repair deficiency (MMR‐D) in approximately 2% of cases, attributed to either germline or somatic mutations. Notably, among this subgroup, four of the five patients treated with ICIs survived beyond 3 years. These long‐term responders harboured either germline (*n* = 3) or somatic (*n* = 1) MMR mutations. Based on these findings, the authors proposed ‘de novo replication repair‐deficient glioblastoma, IDH‐wildtype’ as a distinct molecular subtype of glioma. In contrast, other studies have reported a lack of response to ICIs in MMR‐D GBM [10], highlighting the need for further investigation into predictive biomarkers of therapeutic response. In the present case, although no immunotherapy was administered, the biological context of high TMB may partially explain the prolonged survival in this patient.

Moreover, as reported in LS cases with *MSH6*‐mutant GBM or *POLE* mutations, the case showed ATRX expression, PTEN loss, no *EGFR* amplification nor 10q deletion and methylation subclass referred to pedHGG_RTK1a subtype [7, 8]. ‘pedHGG_RTK1a subtype’ refers to the paediatric‐type high‐grade glioma, receptor tyrosine kinase 1 subgroup, as defined by DNAm profiling. This subgroup is most commonly observed in paediatric populations and is rarely seen in adults, making the molecular classification of this 31‐year‐old patient particularly noteworthy.

Our case AR 10‐037 was 70 years old at the time of diagnosis. Since ageing has been associated with the worst prognosis, his OS is especially remarkable. This LTS case classified as pedHGG clustered sub‐optimally in the MYCN subtype, a subclass comprising H3/IDHwt malignant diffuse gliomas of youth, mostly supratentorial and within the poorest prognosis among the GBM‐IDH1/H3wild subtypes [11]. It is frequently associated with *MYCN* amplifications, often with co‐amplification of the neighbouring *ID2* gene [11], which was absent in our case. Our unique case showed *MDM2* gene amplification. The reciprocal interaction between MYCN and MDM2 is well known, and high MYCN expression requires high MDM2 expression.

Among chromoanagenesis, CT is the most common event in GBM, and it frequently generates gene fusions. Our case presented CT on chromosome 12 harbouring two putative gene fusions:*CPSF6*::*CPM* and *PTPRR*::*RAB3IP* (Figure 2B), never reported in the literature.

In particular, the first chimeric protein *CPSF6::CPM* maintains the entire functional peptidase domain of the *CPM* gene that codifies for an enzyme participating in a variety of processes, such as the control of peptide hormone and growth factor activity at the cell surface, and in the membrane‐localised degradation of extracellular proteins [12].

Ah‐Pine et al. [13] reported that CT mostly involved chr 7, 9 and 12, and in three GBM cases described a gene fusion involving the *CPM* gene with a fusion partner other than *CPSF6* of 52 GBM with CT: no data about the outcome of these cases are published [13].

The second putative chimeric protein partially maintains the protein tyrosine phosphatase domain of the receptor‐type tyrosine‐protein phosphatase R (PTPRR). The latter sequesters mitogen‐activated protein kinases (MAPKs) such as MAPK1, MAPK3 and MAPK14 in the cytoplasm in an inactive form and is preferentially expressed in the brain and lower gastrointestinal tract, so its downregulation might reasonably lead to the development of cancer in these tissues, acting as a tumour‐suppressor gene in cancer development [14].

Although rare cases of *PTPRR* fusion have been reported in GBM [15], the partner fusion gene *RAB3IP* was never described before in GBM patients. Such gene fusions suggest potential biological relevance, though their pathogenic role remains to be elucidated.

In conclusion, the cases we reported here highlight the variability of clinical and molecular characteristics that can be found in long and very long‐term survival GBM patients. Further studies are still needed to identify the biological mechanisms underlying this prognosis. As suggested by recent studies [16], comprehensive molecular characterisation, including DNAm profiling, next‐generation sequencing and RNA sequencing, is expected to make fundamental contributions to this research.

## Author Contributions

**Elena Anghileri:** conceptualization (lead), formal analysis (equal), investigation (equal), visualization (equal), writing – original draft (equal), writing – review and editing (equal). **Evelina Miele:** formal analysis (lead), investigation (equal), methodology (equal), visualization (lead), writing – original draft (equal), writing – review and editing (equal). **Sara Patrizi:** formal analysis (equal), investigation (equal), methodology (equal), writing – original draft (supporting), writing – review and editing (supporting). **Sabina Barresi:** formal analysis (equal), investigation (equal), methodology (equal). **Elisabetta Lazzarini:** formal analysis (equal), investigation (equal), methodology (equal). **Luisa Maddaloni:** formal analysis (equal), investigation (equal), methodology (equal). **Monica Patanè:** formal analysis (equal), investigation (equal). **Lucia Pedace:** formal analysis (equal), investigation (equal), methodology (equal). **Rosina Paterra:** formal analysis (equal), investigation (equal), methodology (equal). **Antonio Silvani:** supervision (equal). **Franco Locatelli:** funding acquisition (equal), supervision (equal). **Stefano Indraccolo:** conceptualization (equal), formal analysis (equal), funding acquisition (equal), investigation (equal), methodology (equal), project administration (equal), supervision (equal), writing – original draft (equal), writing – review and editing (equal). **Bianca Pollo:** conceptualization (equal), formal analysis (equal), funding acquisition (equal), investigation (equal), methodology (equal), project administration (equal), visualization (equal), writing – review and editing (equal).

## Ethics Statement

This study titled ‘Clinical‐neuropathological and molecular study in glioblastoma long‐term survivors (GBM‐LTS)’ was approved by the Clinical Research Ethics Committee (EC) of the Fondazione IRCCS Istituto Neurologico Carlo Besta of Milan FINCB. The protocol was originally approved on 5.4.2017 (Minutes n. 39) and amended and approved on 9.6.2021 (Minutes n. 85) by FINCB EC.

## Conflicts of Interest

The authors declare no conflicts of interest.

## Data Availability

Raw NGS data are deposited in the Sequence Read Archive (SRA) database under BioProject ID PRJNA1078134 (https://www.ncbi.nlm.nih.gov/sra/PRJNA1078134). DNAm raw are deposited in NCBI's Gene Expression Omnibus (GEO; Series accession number GSE230770) and is accessible through https://www.ncbi.nlm.nih.gov/geo/query/acc.cgi?acc=GSE230770 [2]. Other data, including RNASeq data, are available from the corresponding author on reasonable request.
